# National Monitoring of Mosquito Populations and Molecular Analysis of Flavivirus in the Republic of Korea in 2020

**DOI:** 10.3390/microorganisms9102085

**Published:** 2021-10-02

**Authors:** Min-Goo Seo, Hak Seon Lee, Sung-Chan Yang, Byung-Eon Noh, Tae-Kyu Kim, Wook-Gyo Lee, Hee Il Lee

**Affiliations:** Division of Vectors and Parasitic Diseases, Korea Disease Control and Prevention Agency, 187 Osongsaenmyeong2-ro, Osong-eup, Heungdeok-gu, Chungbuk, Cheongju 28159, Korea; koreasmg@korea.kr (M.-G.S.); hslee8510@korea.kr (H.S.L.); npros33@korea.kr (S.-C.Y.); nbudia@korea.kr (B.-E.N.); tkkim80@korea.kr (T.-K.K.); twinleo@korea.kr (W.-G.L.)

**Keywords:** flavivirus, Japanese encephalitis, mosquito, phylogenetic analysis, virus isolation

## Abstract

The Korea Disease Control and Prevention Agency has established centers at 16 locations to screen vector populations and pathogens. The aims of this study were to determine the relative spatiotemporal distributions of mosquitoes that are flavivirus vectors, and to correlate them with instances of flaviviral disease in the Republic of Korea (ROK). We collected 67,203 mosquitoes in traps at 36 collection sites in 30 urban regions and migratory bird habitats in 2020. The trap index was 36.6, and the predominant mosquito species were the *Culex pipiens* complex, *Armigeres subalbatus*, *Aedes albopictus*, *Aedes vexans,* and *Culex tritaeniorhynchus*. The mosquitoes were pooled into 4953 pools to monitor flavivirus infection. We determined that the minimum infection rate of flavivirus was 0.01%. Japanese encephalitis virus (JEV) was detected in only seven pools of *Culex orientalis* from Sangju, and we isolated JVE from two pools. All detected JEV was found to be genotype V by phylogenetic analysis. To the best of our knowledge, this is the first study to isolate genotype V JVE from *Culex orientalis* in the ROK. Subsequent geographical and ecological studies on mosquitoes will help improve our understanding of the relative risk of flavivirus infection. Future studies should analyze mosquito species distribution and improve flavivirus monitoring and long-term surveillance.

## 1. Introduction

Several infectious diseases are transmitted by mosquitoes, and millions of people die annually from them [1]. There are 9 genera and 56 species of mosquitoes in the Republic of Korea [2]. The three main genera are: *Culex* spp., which harbor Japanese encephalitis (JE) virus (JEV) and arbovirus; *Anopheles* spp., which transmit malaria; and *Aedes* spp., which transmit Zika, dengue, yellow, and Chikungunya fevers [3]. It is believed that the annual increases in mean atmospheric temperature, precipitation, and relative humidity associated with global warming could be aggravating the incidence and severity of the aforementioned viral infections by making environmental conditions more conducive to mosquito vector propagation.

Flaviviruses belong to the family Flaviviridae, and the infections they cause have been continuously reported in urban areas throughout subtropical and tropical regions worldwide [4]. From 2016 to 2020, 34 clinical cases of Zika virus infection, 959 cases of dengue fever, and 35 cases of Chikungunya fever were reported in the Republic of Korea. These were all imported rather than indigenous cases [5]. In the Republic of Korea, approximately 1600 cases of JE were reported annually until the 1970s; however, the number of JE cases has markedly decreased since then: only 28, 9, 17, 34, and 7 cases were reported in 2016, 2017, 2018, 2019, and 2020, respectively, because an efficacious vaccine was developed, and a mandatory vaccination program was implemented [6]. To date, there have been no indigenous cases of other flaviviral diseases, such as West Nile or yellow fever, in the Republic of Korea. Nevertheless, the presence of vector mosquitoes increases the probability that this situation might change if mosquito-borne diseases are introduced from abroad [7].

Surveillance systems for mosquito-borne diseases—such as those caused by arboviruses, *Plasmodium* spp., and flaviviruses—have been established, and are actively functional. These monitoring systems assess the relative abundance of mosquitoes as a function of time-series data, and guide public health officials in the application of disease risk mitigation strategies in civilian populations [8]. The Korea Disease Control and Prevention Agency has established local centers to monitor pathogen vector population density related to climate change [9]. To monitor vector control measures and limit the potential impact of pathogens, it is necessary to identify the peak adult mosquito abundance [10]. Here, we studied the spatiotemporal distribution of mosquito populations and the epidemiology of the flaviviruses they harbor in the Republic of Korea. The results of this study could facilitate monitoring and long-term surveillance of vectors and vector-borne pathogens in the interest of global public health.

## 2. Materials and Methods

### 2.1. Ethical Approval

No special permission was required for any collection site, because none of them were located within any national park or protected area. The mosquitoes collected in this study were not members of any endangered species.

### 2.2. Mosquito Population Surveillance

**Figure 1:**
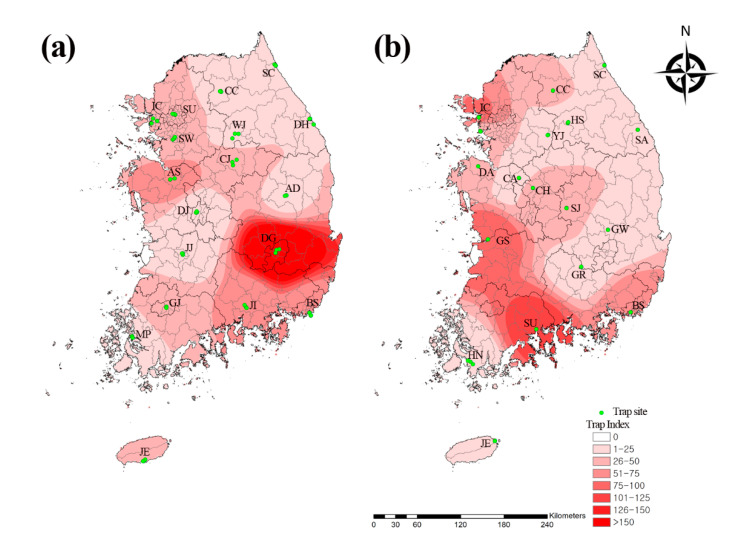
Geographical distribution of mosquitoes in traps at 36 collection sites in (**a**) urban areas and (**b**) migratory bird habitats of the Republic of Korea in 2020. Map color indicates trap index (0 to >161). Collection traps are indicated by green dots. Trap index represents the number of mosquitoes per trap per night. SU: Seoul; IC: Incheon; SC: Sokcho; DH: Donghae; SW: Suwon; CC: Chuncheon; WJ: Wonju; SA: Samcheok; HS: Hoengseong; YJ: Yeoju; CJ: Chungju; AS: Asan; AD: Andong; DG: Daegu; DJ: Daejeon; JJ: Jeonju; CA: Cheonan; CH: Cheongju; DA: Dangjin; SJ: Sangju; GW: Gunwi; GR: Goryeong; GS: Gunsan; GJ: Gwangju; MP: Mokpo; JI: Jinju; BS: Busan; SU: Suncheon; HN: Haenam; JE: Jeju.

Mosquitoes were collected in 2020 from traps at 36 collection sites in 30 regions of the Republic of Korea located in the northern (Seoul, Incheon, Sokcho, Donghae, Suwon, Chuncheon, Wonju, Samcheok, Hoengseong, and Yeoju), central (Chungju, Asan, Andong, Daegu, Daejeon, Jeonju, Cheonan, Cheongju, Dangjin, Sangju, Gunwi, Goryeong, and Gunsan), and southern (Gwangju, Mokpo, Jinju, Busan, Suncheon, and Haenam) areas, as well as the southernmost island (Jeju; Figure 1). Nearly all mosquitoes were captured as adults from 18 collection sites in typical urban residential areas, hills, and parks, as well as 18 other collection sites in migratory bird habitats. Two CDC black-light traps (The John W. Hock Co., Gainesville, FL, USA) and one BG-Sentinel^TM^ trap (Biogents, Regensburg, Germany) baited with dry ice were used over a 24 h period every 2 weeks (first and third weeks of each month) between March and November.

### 2.3. Mosquito Species Identification

All collected mosquitoes were transferred from the traps to Styrofoam coolers containing ice packs. Female mosquitoes were identified and morphologically confirmed using an optical microscope and taxonomic keys [11,12].

### 2.4. Molecular Detection of Flavivirus in Mosquitoes

All mosquitoes except anophelines were used to detect different flavivirus infections, such as those caused by West Nile virus, Zika virus, JEV, yellow fever virus, and dengue fever virus. The mosquitoes were pooled by species, period of study, and collection site. There were 1–30 individuals per pool. The mosquitoes were homogenized with a Precellys^®^ CK28-R lysing kit and bead tube for hard tissue homogenization (Bertin Technologies, Bretonneux, France), as well as a Precellys^®^ evolution homogenizer (Bertin Technologies). Total RNA from the homogenate was extracted with a commercial Bemires^®^ viral RNA extraction kit (IVT7001; InVIRUSTech, Gwangju, Republic of Korea), according to the manufacturer’s protocol.

To detect the non-structural protein 5 (*NS5*) gene of flavivirus, qRT-PCR was performed with a Clear-MD^®^ flavivirus real-time RT-PCR detection kit (IVT-M1001KS; InVIRUSTech). The qRT-PCR conditions for each reaction were 15 min at 50 °C for cDNA synthesis, followed by 10 min at 95 °C for inactivation of reverse transcriptase, 40 cycles of denaturation for 10 s at 95 °C, annealing for 10 s at 60 °C, extension for 10 s at 72 °C, and signal reading for 15 s at 80 °C. After amplification, the qRT-PCR products were subjected to a melting curve analysis to verify each product by its specific melting temperature: denaturation at 95 °C for 30 s, annealing at 60 °C for 1 min, followed by a gradual temperature increase (0.5 °C increments) to 95 °C in 15 s. qRT-PCR reactions were analyzed by Ct values, with Ct ≤ 40 considered positive for flavivirus RNA. When the melting peak of the sample was between 85 °C and 87 °C, it was considered positive. The expected amplicon size of the *NS5* gene was approximately 250 bp. The minimum infection rates (MIRs) were calculated using the following equation:

MIR = number of positive mosquito pools/total number of mosquitoes tested × 100.

### 2.5. RNA Sequencing and Phylogenetic Analysis

The purified RT-PCR products of the *NS5* gene from mosquito pools were obtained with universal M13 forward and reverse amplification primers and sequenced by Macrogen (Seoul, Republic of Korea). Each raw chromatogram of the forward and reverse sequences was visually inspected to detect double peaks and combined into a final whole sequence using CLC Main Workbench 6.9 (CLC Bio; Qiagen, Aarhus, Denmark). The sequences were analyzed using the CLUSTAL Omega v. 1.2.1 (http://www.clustal.org/omega/#Download (accessed on 1 July 2021)) multiple sequence alignment program. The results of the sequence alignments were modified using BioEdit v. 7.2.5 (https://bioedit.software.informer.com/7.2/ (accessed on 1 July 2021)), and analyzed with a similarity matrix. Phylogenetic analysis was performed with MEGA v. 6.0 (https://megasoftware.net (accessed on 1 July 2021)), using the maximum likelihood method with the Kimura 2-parameter model. Tree stability was assessed via bootstrap analysis with 1000 replicates, and the phylogenetic tree was rooted at its midpoint rooting.

### 2.6. Virus Isolation and Purification

Mosquitoes from the positive pools were homogenized, and then centrifuged at 17,000× *g* in a microcentrifuge for 20 min at 4 °C; thereafter, the supernatants were passed through a syringe filter (0.8 µm) and, subsequently, used to inoculate C6/36 cells (ATCC^®^ CRL-1660^TM^; American Type Culture Collection, Manassas, VA, USA). The cells were cultured over a 7-day period at 28 °C, and observed daily to check for the development of cytopathic effect (CPE), which was considered to indicate virus-positive cultures. qRT-PCR was also performed using the cultured samples to confirm viral amplification.

Viral isolation was validated by plaque assay. BHK-21 cells were inoculated at a multiplicity of infection of 0.01, and the CPE of the cells was observed. Tenfold diluted virus supernatants (from 10^−1^ o 10^−6^) were applied to BHK-21 cells in six-well plates and incubated for 1 h at 37 °C. Plates were then overlaid with media containing 75% agarose. After 3–5 days of incubation at 37 °C, overlays were removed, and then plates were covered with crystal violet stain. Virus titers expressed as plaque-forming units (PFUs) were calculated. After viral purification, the JEV envelope (*E*) gene was amplified by RT-PCR using a set of five JEV-specific primers [13] to obtain the complete *E* gene (1500 bp) from isolated viruses.

### 2.7. Geographical and Statistical Analyses

Distribution maps were plotted by interpolation using the inverse distance weighting (IDW) technique in the spatial analyst toolset of ArcGIS v. 9.0 (2004; Environmental Research Systems Institute, Redlands, CA, USA) to compare geographical mosquito distribution. The correlation coefficient method was used to associate *Cx. tritaeniorhynchus* populations with JE cases.

## 3. Results

### 3.1. Prevalence of Mosquito Populations

In total, 67,203 mosquitoes representing 2 subfamilies, 8 genera, and 24 species were collected (Table 1). The mosquitoes were enumerated using the trap index (TI), which is the mean number of female mosquitoes collected per trap per night. Here, the TI was 36.6. We collected 36,537 mosquitoes representing 2 subfamilies, 8 genera, and 19 species from urban areas, and 30,666 mosquitoes representing 2 subfamilies, 8 genera, and 22 species from migratory bird habitats. Of the sampled regions, Daegu (27%; 9855/36,537; TI = 193.2) and Daejeon (1.3%; 479/36,537; TI = 9.4) had the highest and lowest numbers of collected mosquitoes in the urban areas, respectively, while Suncheon (17.8%; 5472/30,666; TI = 107.3) and Samcheok (0.5%; 159/30,666; TI = 3.1) had the highest and lowest numbers of collected mosquitoes in the migratory bird habitats, respectively (Table 1). The urban areas had a higher incidence of mosquitoes (54.4%) than the migratory bird habitats (45.6%). The TI of the mosquitoes in the urban areas was 1.2-fold higher (39.8) than that of those in the migratory bird habitats (33.4) (Figure 1).

The *Cx. pipiens* complex (37.8%; 25,395/67,203; TI = 13.8) was the most prevalent mosquito species, followed by *Ar. subalbatus* (13.8%; 9305/67,203; TI = 5.1), *Ae. albopictus* (13.1%; 8789/67,203; TI = 4.8), *Ae. vexans* (11.0%; 7393/67,203; TI = 4), *Cx. tritaeniorhynchus* (9.3%; 6221/67,203; TI = 3.4), *Anopheles spp*. (4.9%; 3282/67,203; TI = 1.8), *Och. koreicus* (3.5%; 2375/67,203; TI = 1.3), *Cx. orientalis* (2.0%; 1370/67,203; TI = 0.7), *Och. dorsalis* (2.0%; 1324/67,203; TI = 0.7), *Man. uniformis* (1.0%; 698/67,203; TI = 0.4), and *Cx. inatomii* (0.7%; 481/67,203; TI = 0.3) (Table 1). Each of the remaining 13 species accounted for < 1% of the total number of mosquitoes collected.

The main incidence of mosquitoes occurred from the 23rd week (first week of June; 6027/67,203; TI = 55.8) to the 40th week (first week of October; 4014/67,203; TI = 37.2) of the year (Table 2). The mosquito populations suddenly increased in the 23rd week, and the first peak was observed in the 29th week (third week of July; 11.2%; 7560/67,203; TI = 70.0). The mosquito populations slightly decreased in the 32nd week (first week of August; 8.6%; 5809/67,203; TI = 53.8), and the maximum peak was observed in the 36th week (first week of September; 13.6%; 9170/67,203; TI = 84.9). Thereafter, the mosquito populations steadily decreased.

**Figure 2:**
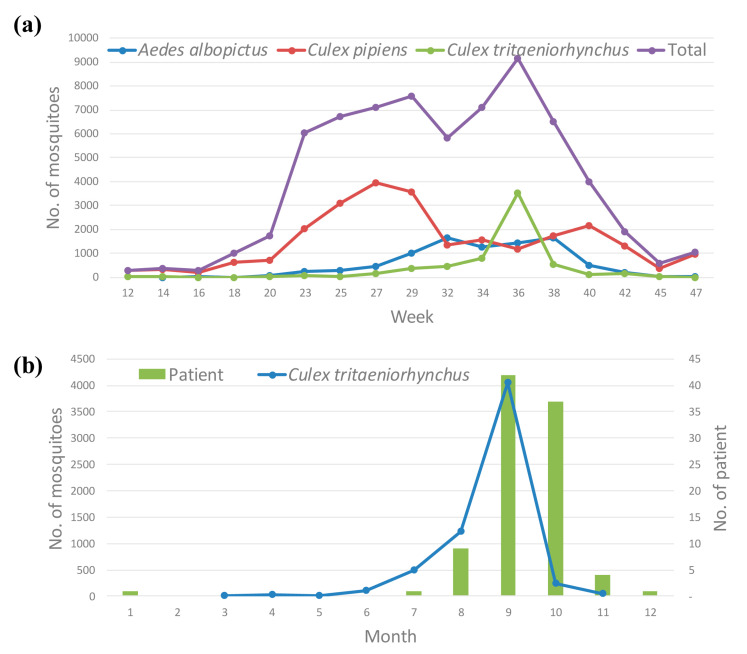
(**a**) Temporal distribution of the populations of the three main mosquito species carrying flavivirus, individually and combined; (**b**) monthly fluctuations in accumulated Japanese encephalitis cases from 2016 to 2020, and *Culex* *tritaeniorhynchus* harboring Japanese encephalitis virus in the Republic of Korea in 2020.

Of the major flavivirus vectors, the highest numbers of the *Cx. pipiens* complex, *Ae. albopictus*, and *Cx. tritaeniorhynchus* were collected in the 27th week (first week of July; 15.6%; 3960/25,395; TI = 36.7), 32nd week (first week of August; 18.8%; 1652/8789; TI = 15.3), and 36th week (first week of September; 56.7%; 3530/6221; TI = 32.7), respectively (Figure 2a).

Between 2016 and 2020, JE cases started to appear in July, and reduced by December. Most cases were reported between September and October (83.2%; 79/95) (Figure 2b). The numbers of *Cx. tritaeniorhynchus* collected were recorded primarily between August and September (85%; 5289/6221) in 2020. There was consistency between the number of JE cases and the period 1 month after *Cx. tritaeniorhynchus* incidence. A correlation coefficient analysis showed satisfactory agreement between *Cx. tritaeniorhynchus* incidence and JE cases (0.9545; *p* = 0.0031; 95% confidence interval = 0.6343–0.9952).

**Figure 3:**
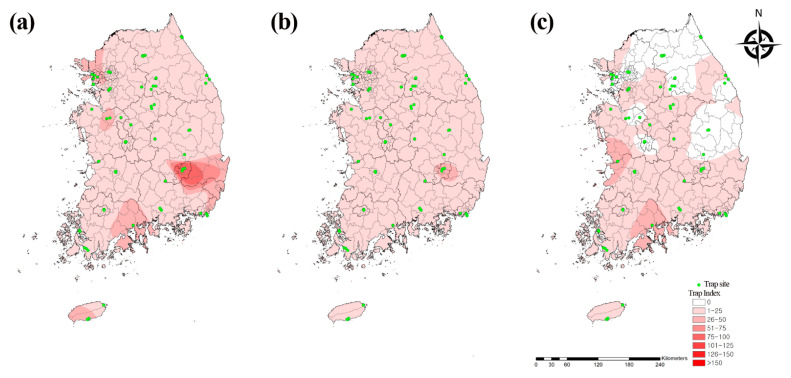
Geographical distribution of three major flavivirus vector mosquito species. (**a**) *Culex pipiens* complex, (**b**) *Aedes albopictus*, and (**c**) *Culex tritaeniorhynchus* collected from traps at 36 collection sites in the Republic of Korea in 2020. Map color indicates TI (0 to >160). Collection traps are indicated by green dots. TI shows the number of mosquitoes per trap per night.

We analyzed the collection data using the IDW interpolation tool in ArcGIS (https://www.arcgis.com/index.html (accessed on 1 July 2021)) to assess the geographical distribution of the mosquitoes in the Republic of Korea. We initially plotted distribution maps based on the numbers of each of the three major flavivirus vector mosquito species (Figure 3). The *Cx. pipiens* complex and *Ae. albopictus* were distributed nationwide, whereas *Cx. tritaeniorhynchus* was distributed mainly in the southern and western regions of the country.

### 3.2. Virus Isolation and Purification

Of the 67,203 mosquitoes sampled, 63,662 were categorized into 4953 pools, and the flavivirus MIR was 0.01% (seven pools/63,662 mosquitoes; Table 2). Seven *NS5* pools were identified in *Cx. orientalis* localized to migratory bird habitats (MIR = 0.03%; 7/27,811) in Sangju (MIR = 0.3%; 7/2407) during the third week of August (MIR = 0.5%; 1/221), first week of September (MIR = 0.7%; 2/291), and third week of September (MIR = 0.8%; 4/481).

**Figure 4:**
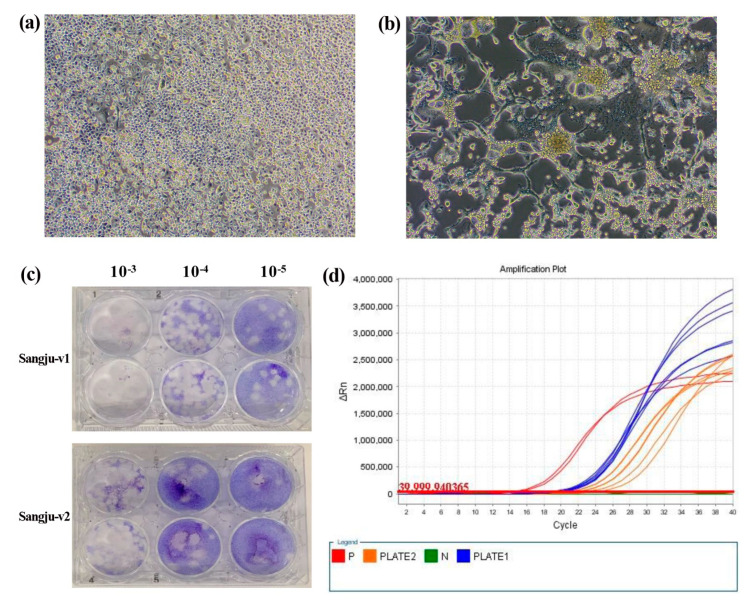
Results of Japanese encephalitis virus isolation: (**a**) Control C6/36 cells and (**b**) C6/36 cells 4 days after infection with positive mosquito sample showing cytopathic effects (200 × magnification). (**c**) Plaque phenotypes in BHK-21 cells. (**d**) Amplification curve of isolated Japanese encephalitis virus. P: positive sample; Plate1: Sangju-v1; Plate 2: Sangju-v2; N: negative sample.

Of the seven JEV-positive *NS5* pools, only two viruses were isolated during the third week of September. A viral isolate was identified that caused CPE in C6/36 cells, and which was characterized by aggregation and marked syncytia by day 4 post-infection (Figure 4a,b). Of the isolated viruses, mean virus titers were 2.42 × 10^6^ pfu/mL and 4.75 × 10^5^ pfu/mL, respectively (Figure 4c), and mean qRT-PCR Ct values were 19.7 and 22.1, respectively (Figure 4d). To confirm the viral genotype, we amplified the JEV-specific *E* gene. These isolated viruses were donated to the Gene Resource Bank of the Republic of Korea.

### 3.3. Molecular and Phylogenetic Analyses

**Figure 5:**
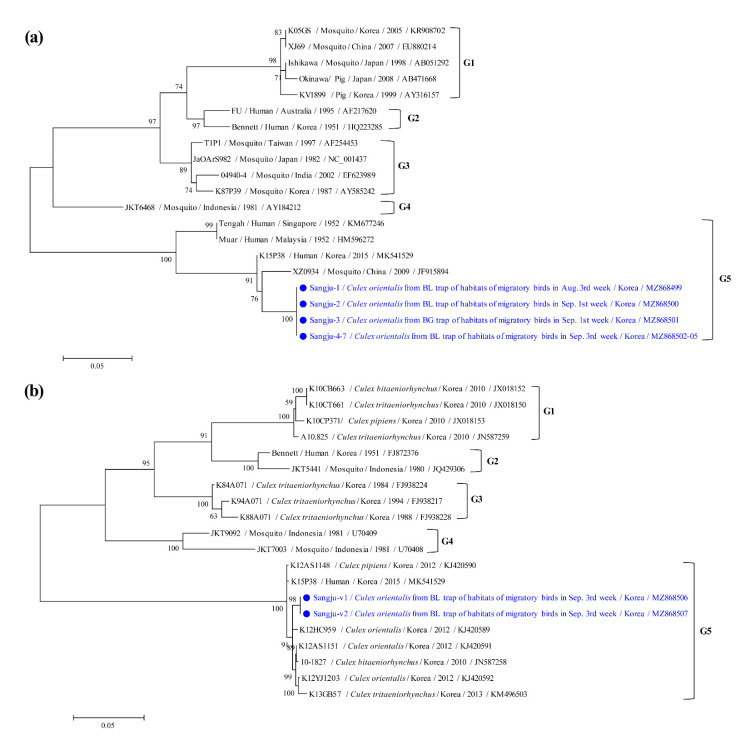
Phylogenetic tree of Japanese encephalitis virus (JEV) based on (**a**) non-structural protein 5 (*NS5*) gene sequences and (**b**) envelope (*E*) gene sequences. The maximum likelihood method was used to plot the tree with the Kimura 2-parameter model. Closed circles and blue letters indicate sequences detected here. GenBank accession numbers are shown. JEV genotypic groups are indicated. Branch numbers indicate bootstrap support levels (1000 replicates). The phylogenetic tree is rooted at its midpoint rooting. Scale bar displays substitution numbers for each nucleotide.

A phylogenetic analysis showed that *NS5* (Figure 5a) and *E* (Figure 5b) in JEV were clustered with previously documented sequences. Seven mosquito *NS5* sequences and two viral *E* sequences comprised genotype V.

The seven JEV strains of *NS5* detected here shared 100% identity. Each sequence shared 91.1–97.9% identity with the genotype V strains reported for the JEV isolates in GenBank. The two JEV strains of *E* found in this study shared 100% identity. Each sequence shared 99.1–99.9% identity with the genotype V strains reported for the JEV isolates in GenBank. Representative sequences reported in the present study were submitted to GenBank under accession numbers MZ868499–MZ868507.

## 4. Discussion

Several climate variables, such as temperature and precipitation, have significantly changed because of global warming. These are the main driving forces of vector-borne diseases, because they can modify vector development [7] by influencing pathogen fate, transmission, stability, reproduction rates, and environmental variability. Emerging mosquito-borne diseases continue to threaten public health. It is necessary to monitor the influx of transmission vectors, such as *Ae. albopictus*, which transmits dengue, Chikungunya, and Zika fevers, as well as the *Cx. pipiens* complex, which transmits West Nile fever [14]. Both *Ae. albopictus* and *Cx. pipiens* are commonly distributed throughout the Republic of Korea. A recent study showed that *Ae. albopictus* populations are widely distributed in the urban areas of the Republic of Korea. Nevertheless, the flaviviruses they harbor—including Zika, Chikungunya, and dengue fever viruses—were not detected in 2016 [15].

To monitor mosquito population distribution, we collected mosquitoes from traps in urban residential areas, hills, and parks, which are accessible to humans, as well as from traps in migratory bird habitats with the potential to introduce foreign mosquito-borne diseases. Mosquitoes were surveyed and analyzed every 2 weeks from the 12th to the 47th weeks (third week of March to the third week of November) of 2020. The total number of mosquitoes was lower in the summer of 2020 than in the summer of 2019 because the former had low average temperatures, a monsoon season, a high number of precipitation days, and a large cumulative precipitation volume [16].

We identified the geographical and temporal distribution of the mosquito species. The *Cx. pipiens* complex and *Ae. albopictus* were distributed throughout the country, whereas *Cx. tritaeniorhynchus* was localized mainly in southern regions, including Gunsan (37.2%) and Suncheon (34.7%). Among the regions, Daegu (14.7%), Incheon (11.5%), Busan (9.3%), Suncheon (8.1%), and Gunsan (6.7%) accounted for more than 50% of the total mosquito population distribution and endemicity. Geographical differences in mosquito populations may be influenced by both ecological and environmental factors. Daegu had a trap site in an urban park with outdoor human activity, which had the highest mosquito distribution in the present study. As this park is a major pest control point, ongoing insect management is required to mitigate mosquito-borne disease transmission to humans. However, Incheon, Suncheon, and Gunsan were major migratory bird habitats and had high mosquito distribution. These regions should be continuously monitored for mosquito-borne diseases (e.g., West Nile fever), which are transmitted by migratory birds.

Of the four major mosquito species associated with mosquito-borne diseases, the *Cx. pipiens* complex (37.8%) was the most prevalent, followed by *Ae. albopictus* (13.1%), *Cx. tritaeniorhynchus* (9.3%), and *Anopheles spp*. (4.9%). The *Cx. pipiens* complex (48.8%) and *Ae. albopictus* (18.0%) were prevalent in urban areas, whereas the *Cx. pipiens* complex (24.7%), *Cx. tritaeniorhynchus* (18.4%), *Anopheles spp*. (9.1%), and *Ae. albopictus* (7.2%) were predominant in migratory bird habitats. The distribution of the JE vector *Cx. tritaeniorhynchus* was greater in migratory bird habitats than in urban areas. By contrast, the distribution of *Ae. albopictus* was greater in hills and urban parks than in residential areas [15]. On the other hand, *Ar. subalbatus* was the second most prevalent mosquito species in this study; it is the transmission vector of JE [17] and Zika fever [18]. Moreover, *Ae. vexans*, which is the transmission vector of West Nile [19] and Zika fevers [20], was the fourth most prevalent mosquito species in this study. Since *Ar. subalbatus* and *Ae. vexans* are widely distributed throughout the Republic of Korea, they must be considered and included in the prevention and control systems of mosquito-borne diseases.

Adult mosquito distribution depends on the seasonal climate and ecological factors affecting larval growth and development. In the Republic of Korea, the abundance of mosquitoes increases from May to late August or early September, and decreases from October onwards when mean temperatures reduce [8]. In the present study, the mosquito populations were prevalent between the first week of June and the first week of October. Mosquitoes are the main flavivirus vectors, and their prevalence varies by species. Here, the *Cx. pipiens* complex was normally distributed throughout the entire surveillance period, and its highest prevalence was in the 27th week (first week of July). *Ae. albopictus* was distributed primarily between August and September, and its highest prevalence was in the 32nd week (first week of August). In 2016, *Ae. albopictus* was mainly prevalent between August and September [15]. *Cx. tritaeniorhynchus* had the highest prevalence in the 36th week (first week of September) of 2020; it also had the highest prevalence in the 36th week (first week of September) of 2019 and the 37th week (second week of September) of 2020 [16]. This difference could be explained by species-specific temperature requirements for eclosion and development from the larval to the adult stage [8]. Hence, it is important to identify the temperature ranges at which each mosquito species shows maximum abundance. This information can be effectively used for vector control. The mean atmospheric temperatures for the maximum abundance of the *Cx. pipiens* complex, *Cx. tritaeniorhynchus*, and *Ae. albopictus* were 22.6 °C, 24.3 °C, and 24.6 °C, respectively [8]. Depending on the mosquito species and stage of development, the upper and lower atmospheric temperature limits are in the ranges of 30–35 °C and 5–10 °C, respectively [8].

JE is distributed in the tropical and temperate areas of eastern and southern Asia, where paddy fields are irrigated. Rice cultivation provides a suitable habitat for paddy-breeding mosquitoes, such as *Cx. tritaeniorhynchus*, which is the main JE vector in most parts of Asia. Nevertheless, other *Culex* spp. are secondary or regional JE vectors [21]. Since the aforementioned species share the same ecological niche (i.e., irrigated paddy fields), JE is highly prevalent in rural areas [17]. It is normally prevalent in the southern regions of the Republic of Korea, where *Cx. tritaeniorhynchus* predominates. Since 2010, however, JE’s prevalence has also increased in the northern areas of the Republic of Korea [22]. This finding is consistent with fact that genotype V JEV has been detected in various mosquito species, including *Cx. tritaeniorhynchus*, *Cx. orientalis*, and the *Cx. pipiens* complex [13]. In the present study, JEV was detected in only seven *Cx. orientalis* pools. The number of *Cx. orientalis* collected in the urban areas (69.0%; TI = 6.5) and migratory bird habitats (28.7%; TI = 4.6) was the highest in the 34th week (third week of August). JEV was also detected in *Cx. orientalis* in the migratory bird habitats of Sangju during August and September. Although *Cx. orientalis* harbors JEV, its role as a JE vector in humans is unknown. Thus, further studies are needed in order to analyze the transmission of JE infection via other potential vectors (e.g., *Cx. orientalis*).

The number of JE cases displayed a seasonal pattern, and most cases were reported between August and November [5]. We compared temporal distributions of mosquito populations against the incidence of JE in human patients. In 2016–2020, JE cases started to appear in July, and ended in December. As *Cx. tritaeniorhynchus* populations began to increase in July, and peaked in September, the number of human JE cases increased from August to October. As there was a sudden decrease in *Cx. tritaeniorhynchus* populations in October, the number of human JE cases decreased in November. Therefore, high *Cx. tritaeniorhynchus* density might affect the annual incidence of JE. We found a strong correlation (0.9545) between *Cx. tritaeniorhynchus* distribution at 1-month intervals and JE incidence.

We performed molecular detection and phylogenetic analyses of JEV in mosquitoes. We expected that the highly endemic regions would show relatively higher *Cx. tritaeniorhynchus* population densities than other regions. However, we found no correlation between the relative rates of JE cases and mosquito population densities. Even though Suwon had the highest JE incidence in 2020 (28.6%; n = 2), it also had the lowest *Cx. tritaeniorhynchus* population density (TI = 0.2). By contrast, Gunsan had the highest *Cx. tritaeniorhynchus* population density (TI = 45.4), but no cases of JE in 2020. These discoveries suggest that *Cx. tritaeniorhynchus* population density alone does not explain JE epidemicity, and that other geographical and ecological factors contribute to JE incidence. In Sangju, we only detected JEV in *Cx. orientalis*, and there were no reported cases of JE in 2020. The densities of *Cx. tritaeniorhynchus* populations were lower (TI = 0.2) than those of *Cx. orientalis* populations (TI = 7.7) in Sangju.

The *E* gene of JEV plays a major role in the pathogenesis of encephalitis [23]. Here, the *NS5* gene appeared in seven genotype V pools in JEV. The virus was isolated from only two pools, and the *E* gene of JEV was analyzed. To the best of our knowledge, the present study is the first to isolate genotype V JEV from *Cx. orientalis* in the Republic of Korea. However, the bionomics of this mosquito species would hinder it from causing a large JE outbreak. Its larvae live only in fresh water, such as slowly moving mountain streams and ponds in the Republic of Korea, and its adults do not feed on humans [24]. In other studies conducted in the Republic of Korea, JEV genotypes I and V were detected in *Cx. tritaeniorhynchus* and *Cx. bitaeniorhynchus*, respectively, between 2008 and 2010 [25]; genotype I was found in the *Cx. pipiens* complex, *Cx. tritaeniorhynchus*, and *Cx. bitaeniorhynchus* in 2010 [26]; genotype V was detected in *Cx. orientalis* and the *Cx. pipiens* complex in 2012 [13]; genotype V was isolated from humans in 2015 [22] and 2018 [27]; and genotype V was identified in the *Cx. pipiens* complex between 2016 and 2018 [28]. The genotype V *E* gene sequences showed 99.1–99.9% identity between mosquitoes and humans in the Republic of Korea. Estimation of the origin of the *E* gene of JEV genotype V indicated that the XZ0937 strain of *Cx. tritaeniorhynchus* in Tibet (China) in 2009 was an ancestor of the JEV genotype V strain in the Republic of Korea [22].

Genotype III was the dominant global strain until the latter part of the 20th century. Thereafter, a JEV genotype shift from type III to type I was reported in many areas, with the latter becoming the dominant strain in several countries [29]. By contrast, genotype V was a rare strain first detected in encephalitis patients in Malaysia and Singapore in 1952. Patients with JEV genotype V exhibit clinical manifestations including vomiting, neck stiffness, high-grade fever, headache, disturbed consciousness, and deep coma with rapid progression to death by respiratory failure. Genotype V was also reported in *Cx. tritaeniorhynchus* in Tibet (China) in 2009 [30]. In the Republic of Korea, the JEV genotype III strain predominated until genotype I was isolated in 1994. Since that time, only genotype I strains had been isolated until 2010 [31]. The predominant JEV genotype from mosquitoes changed from I to V in 2010 in the Republic of Korea [32]. Hence, the number of infected adult patients has increased accordingly [22,27], and the patients with JEV genotype V often present with mild symptoms—such as headache, fever, nausea, and apathy—in the Republic of Korea [22]. Genotype V is seldom reported in other countries, whereas several cases have been recently reported in the Republic of Korea. However, there is limited documentation on the pathogenicity of the genotype V strain, and it is unknown whether the currently available genotype-III-based vaccines are effective against genotype V. Another study reported that existing JEV genotype III vaccines have limited protective efficacy against JEV genotype V [32]. Therefore, further research is required in order to assess the efficiency of existing JE vaccines against genotype V JEV. Moreover, comparison with JEV genotypes I–IV revealed the insertion of three nucleotides (encoded with a serine residue) in the *NS4A* gene of JEV genotype V; nucleotide insertion was also detected downstream of the open reading frame stop codon in 3′-untranslated regions. In addition, numerous amino acid mutations were observed in three functional domains of the *E* gene of JEV genotype V [33]. In the present study, we were unable to determine the impact of the emergence of this novel genotype V on the present JE outbreak in the Republic of Korea. Thus, in-depth studies on genotype V are necessary in order to identify its potential vectors and impact on JE outbreaks in humans. Additionally, further studies are needed in order to assess the virulence and pathogenicity of the isolated JEV genotype V in this study.

In summary, in the present study, we surveyed the spatiotemporal distribution of mosquito populations in the Republic of Korea, and performed a molecular analysis on the flaviviruses that they harbor. We identified broadly distributed mosquito species, as well as the presence of JEV genotype V in those that were localized to migratory bird habitats. The seasonal distributions of the mosquitoes collected in this study reflected their life cycles and ecological factors. Monitoring of patients with possible mosquito-borne diseases should be prioritized in order to reduce potential viral transmission to resident populations. Future geographical and ecological studies on mosquitoes will improve our understanding of flavivirus infection risk in the Republic of Korea. The distribution of mosquito species must be analyzed, and flavivirus monitoring and long-term surveillance must be improved.

## Figures and Tables

**Table 1:** Geographical distribution of mosquito species collected from traps in the Republic of Korea in 2020.

**Regions**	No. of Collected Mosquitoes																										
	Cx.pip	Ae. albop	Cx. tri	Ar. sub	Ae. vex	Anopheles spp.	Och. kor	Cx. ori	Och. dor	Man. uni	Cx. ina	Och. tog	Cx. bit	Ae. lin	Coq. och	Cx. vag	Och. hat	Tri. bam	Ae. albos	Och. jap	Ae. eso	Ae. fla	Och. ore	Others	Total	%	TI
Urban areas																											
Seoul	883	563	1	112	17	4	269	0	0	0	0	0	0	0	0	1	0	0	0	0	0	0	0	0	1850	5.1	36.3
Incheon	2095	173	0	0	33	10	63	1	5	0	0	0	2	0	0	0	0	0	1	0	0	0	0	0	2383	6.5	46.7
Sokcho	229	79	0	220	12	8	26	1	0	0	0	0	0	0	0	0	0	0	0	0	0	0	0	0	575	1.6	11.3
Donghae	636	196	65	208	4	2	57	0	0	0	0	4	0	0	0	1	0	1	0	0	0	0	0	1	1175	3.2	23.0
Suwon	1116	33	8	143	64	26	84	7	0	1	8	0	2	0	0	0	0	0	0	0	0	0	0	0	1492	4.1	29.3
Chuncheon	148	334	0	240	48	98	72	4	0	0	0	0	0	0	0	0	0	1	0	0	0	0	0	0	945	2.6	18.5
Wonju	66	110	0	226	287	13	103	2	0	0	0	0	0	0	0	0	0	0	0	0	0	0	0	0	807	2.2	15.8
Chungju	268	158	33	1250	201	2	316	59	0	0	0	0	2	0	0	0	0	0	0	0	0	0	0	0	2289	6.3	44.9
Asan	1358	949	3	218	191	25	40	419	0	0	0	0	0	0	0	0	0	0	0	0	0	0	0	0	3203	8.8	62.8
Andong	638	325	0	56	2	10	15	3	0	0	0	0	0	0	0	0	0	0	0	0	0	0	0	0	1049	2.9	20.6
Daegu	4109	1411	25	4002	35	69	204	0	0	0	0	0	0	0	0	0	0	0	0	0	0	0	0	0	9855	27	193.2
Daejeon	247	5	1	61	17	131	9	1	0	0	0	0	0	0	0	1	0	0	5	1	0	0	0	0	479	1.3	9.4
Gwangju	1208	321	364	20	28	36	68	1	1	55	0	0	1	0	1	0	0	0	0	0	0	0	0	0	2104	5.8	41.3
Jeonju	493	38	26	31	23	19	17	4	0	21	8	0	1	0	0	0	0	0	0	0	0	0	0	0	681	1.9	13.4
Mokpo	265	161	18	189	0	26	0	0	8	0	0	5	2	0	0	0	0	0	0	0	0	0	0	0	674	1.8	13.2
Jinju	802	1275	10	123	113	21	341	4	0	0	0	0	0	0	0	0	8	0	0	0	0	0	0	0	2697	7.4	52.9
Busan	1950	360	6	195	2	0	11	0	0	0	0	212	0	0	0	0	0	0	0	0	0	0	0	0	2736	7.5	53.6
Jeju	1318	87	7	117	0	2	9	0	0	0	1	1	1	0	0	0	0	0	0	0	0	0	0	0	1543	4.2	30.3
Subtotal	17,829	6578	567	7411	1077	502	1704	506	14	77	17	222	11	0	1	3	8	2	6	1	0	0	0	1	36,537	100	39.8
Migratory bird habitats																											
Samcheok	3	85	0	61	1	1	3	1	0	0	0	1	0	0	0	0	0	3	0	0	0	0	0	0	159	0.5	3.1
Incheon	3030	13	53	2	578	210	8	4	1293	4	101	5	6	47	6	0	0	0	0	0	0	0	0	0	5360	17.5	52.5
Hoengseong	102	167	1	219	143	1	5	1	0	0	0	0	0	0	0	0	0	1	0	0	0	0	0	0	640	2.1	12.5
Sokcho	166	27	0	1	10	2	5	2	11	0	0	0	0	0	0	0	0	0	0	0	0	0	0	0	224	0.7	4.4
Yeoju	94	120	67	68	489	80	23	12	0	0	0	0	1	0	0	0	0	0	0	0	0	0	0	0	954	3.1	18.7
Chuncheon	114	499	0	435	134	42	146	34	0	0	0	0	2	0	0	0	0	1	0	0	0	0	0	1	1408	4.6	27.6
Cheonan	39	23	0	19	56	10	4	10	0	0	0	4	1	0	0	0	0	0	0	3	0	0	0	0	169	0.6	3.3
Cheongju	534	118	134	326	374	36	162	87	0	0	0	0	3	0	0	0	0	0	0	0	0	0	0	0	1774	5.8	34.8
Dangjin	274	165	50	85	911	74	33	175	0	3	0	0	2	0	0	15	0	0	0	0	0	0	0	0	1787	5.8	35.0
Sangju	149	174	10	251	1380	71	16	395	0	2	0	0	23	0	0	0	0	5	0	0	1	0	1	0	2478	8.1	48.6
Gunwi	82	90	0	73	10	15	49	21	0	0	0	0	0	0	0	0	0	0	0	0	0	0	0	0	340	1.1	6.7
Goryeong	38	151	48	147	90	145	18	11	0	0	0	0	0	0	0	1	22	0	0	0	0	1	0	0	672	2.2	13.2
Gunsan	436	22	2316	14	380	211	122	60	1	438	361	1	49	23	50	18	0	0	0	1	0	0	0	0	4503	14.7	88.3
Suncheon	1631	166	2161	28	539	856	27	10	5	43	0	0	4	2	0	0	0	0	0	0	0	0	0	0	5472	17.8	107.3
Busan	836	28	788	15	1215	428	19	41	0	131	0	1	0	0	7	0	0	0	0	0	0	0	0	0	3509	11.4	68.8
Haenam	13	295	19	11	3	22	0	0	0	0	0	0	0	0	0	0	0	0	0	0	0	0	0	0	363	1.2	7.1
Jeju	25	68	7	139	3	576	31	0	0	0	2	3	0	0	0	0	0	0	0	0	0	0	0	0	854	2.8	16.7
Subtotal	7566	2211	5654	1894	6316	2780	671	864	1310	621	464	15	91	72	63	34	22	10	0	4	1	1	1	1	30,666	100	33.4
Total	25,395	8789	6221	9305	7393	3282	2375	1370	1324	698	481	237	102	72	64	37	30	12	6	5	1	1	1	2	67,203	-	36.6

TI, trap index (no. of mosquitoes collected /no. of installed traps/nights); Ae. albop: *Aedes albopictus*; Ae. albos: *Aedes alboscutellatus*; Ae. eso: *Aedes esoensis*; Ae. fla: *Aedes flavopictus*; Ae. lin: *Aedes lineatopennis*; Ae. vex: *Aedes vexans*; Ar. sub: *Armigeres subalbatus*; Coq. och: *Coquillettidia ochracea*; Cx. bit: *Culex bitaeniorhynchus*; Cx. ina: *Culex inatomii*; Cx. ori: *Culex orientalis*; Cx. pip: *Culex pipiens* complex; Cx. tri: *Culex tritaeniorhynchus*; Cx. vag: *Culex vagans*; Man. uni: *Mansonia uniformis*; Och. dor: *Ochlerotatus dorsalis*; Och. hat: *Ochlerotatus hatorii*; Och. jap: *Ochlerotatus japonicas*; Och. kor: *Ochlerotatus koreicus*; Och. ore: *Ochlerotatus oreophilus*; Och. tog: *Ochlerotatus togoi*; Tri. bam: *Tripteroides bambusa*; Others: unidentified.

**Table 2:** Temporal flavivirus distribution and infection by mosquitoes in the Republic of Korea in 2020.

Species	March	April		May		June		July		August		September		October		November		No. of Mosquitoes				TI
	3rd wk	1st wk	3rd wk	1st wk	3rd wk	1st wk	3rd wk	1st wk	3rd wk	1st wk	3rd wk	1st wk	3rd wk	1st wk	3rd wk	1st wk	3rd wk	Collected (%)	Tested	Pools	Positive Pools (MIR)	
Cx. pip	263	316	189	618	701	2013	3096	3960	3583	1369	1560	1188	1731	2144	1319	371	974	25,395 (37.8)	25,209	1618	0	13.8
Ae. albop	5	0	42	1	77	228	289	444	988	1652	1281	1423	1640	474	216	24	5	8789 (13.1)	8747	671	0	4.8
Cx. tri	5	25	0	4	14	73	31	148	348	460	774	3530	525	97	140	47	0	6221 (9.3)	6217	356	0	3.4
Cx. ori	5	2	5	15	1	21	82	102	101	112	597	208	113	2	3	0	1	1370 (2.0)	1370	207	7 (0.5)	0.7
Others	6	12	42	362	949	3692	3227	2465	2540	2216	2897	2821	2513	1297	212	118	59	25,428 (37.8)	22,119	2101	0	13.8
Total	284	355	278	1000	1742	6027	6725	7119	7560	5809	7109	9170	6522	4014	1890	560	1039	67,203	63,662	4953	7 (0.01)	36.6

wk: week; TI: trap index (no. of mosquitoes collected/no. of installed traps/nights); MIR: minimum infection rate (no. of positive mosquito pools/total no. of mosquitoes tested × 100); Ae. albop: *Aedes albopictus*; Cx. ori: *Culex orientalis*; Cx. pip: *Culex pipiens* complex; Cx. tri: *Culex tritaeniorhynchus*; Others: other species.

## Data Availability

Data supporting the conclusions of this article are included within the article. The newly generated sequences were submitted to the GenBank database under the accession numbers MZ868499–MZ868507. The datasets used and/or analyzed during the present study are available from the corresponding author upon reasonable request.
